# Pantoprazole, an FDA-approved proton-pump inhibitor, suppresses colorectal cancer growth by targeting T-cell-originated protein kinase

**DOI:** 10.18632/oncotarget.7984

**Published:** 2016-03-08

**Authors:** Xiaoyu Zeng, Lin Liu, Mengzhu Zheng, Huimin Sun, Juanjuan Xiao, Tao Lu, Guangqian Huang, Pianpian Chen, Jianmin Zhang, Feng Zhu, Hua Li, Qiuhong Duan

**Affiliations:** ^1^ Department of Biochemistry and Molecular Biology, School of Basic Medicine, Huazhong University of Science and Technology, Wuhan, Hubei, 430030, PR China; ^2^ School of Pharmacy, Tongji Medical College, Huazhong University of Science and Technology, Wuhan, Hubei, 430030, PR China; ^3^ Department of Urology, Xijing Hospital, the Fourth Military Medical University, Xi'an, Shaanxi, 710032, PR China; ^4^ Department of Neurobiology, School of Basic Medicine, Huazhong University of Science and Technology, Wuhan, Hubei, 430030, PR China; ^5^ School of Traditional Chinese Materia Medica, Shenyang Pharmaceutical University, Shenyang, Liaoning, 110016, PR China

**Keywords:** pantoprazole, PPI, TOPK, cell transformation, colon carcinoma

## Abstract

T-cell-originated protein kinase (TOPK) is highly expressed in several cancer cells and promotes tumorigenesis and progression, and therefore, it is an important target for drug treatment of tumor. Pantoprazole (PPZ) was identified to be a TOPK inhibitor from FDA-approved drug database by structure based virtual ligand screening. Herein, the data indicated that pantoprazole inhibited TOPK activities by directly binding with TOPK *in vitro* and *in vivo*. *Ex vivo* studies showed that pantoprazole inhibited TOPK activities in JB6 Cl41 cells and HCT 116 colorectal cancer cells. Moreover, knockdown of TOPK in HCT 116 cells decreased their sensitivities to pantoprazole. Results of an *in vivo* study demonstrated that i.p. injection of pantoprazole in HCT 116 colon tumor-bearing mice effectively suppressed cancer growth. The TOPK downstream signaling molecule phospho-histone H3 in tumor tissues was also decreased after pantoprazole treatment. In short, pantoprazole can suppress growth of colorectal cancer cells as a TOPK inhibitor both *in vitro* and *in vivo*.

## INTRODUCTION

Recently, cancer drug development has shifted from traditional cytotoxic drugs to agents that target the special molecular pathology, driving the progression of ‘personalized’ medicine [1]. The typical representative for this alternative approach for the treatment of cancer is imatinib, a BCR-Abl tyrosine kinase inhibitor, which left a deep impression on people [2]. For targeted cancer therapy, small-molecule inhibitors fall broadly into four categories. They are kinase inhibitors, chaperone inhibitors, histone deacetylase inhibitors and inhibitors of protein-protein interactions. In this study, we devote ourselves to studying T-cell-originated protein kinase (TOPK) inhibitors.

TOPK is a serine-threonine kinase, a member of MAPKK family. TOPK was confirmed to be highly expressed in multiple types of cancers and associated with poor prognosis, such as lymphoma, leukemia, melanoma, colorectal, breast and lung cancers, and cholangiocarcinoma [3–10]. Also, TOPK plays an important role in tumor development and progression [11–13]. Above all, TOPK exhibits high expression levels in cancer tissues but only low expression levels in normal tissues [11]. These suggest that TOPK might be an excellent drug target for cancer chemotherapy. However, there is still no TOPK inhibitor in clinic use. There are two TOPK inhibitors, HI-TOPK-032 [14] and OTS964 [15], reported in 2012 and 2014 respectively. However, because of the solubility and toxicity of the two compounds, there would be a long way for them to be used clinically.

In this study, structure based virtual ligand screening method was employed to screen the FDA-approved drug database and pantoprazole (PPZ) was identified as a TOPK inhibitor.

## RESULTS

### Virtual ligand screening identifies the binding of pantoprazole to TOPK

Due to the absence of the crystal structure of TOPK or its closely related homologue, a homology model of human TOPK was constructed by using the X-ray structure of the IRAK-4 kinase (PDB code: 2NRU) as the template, which has 29% of sequence identities to the human TOPK and 2.0 Å resolution of the structure. The model generated was used for subsequent molecular docking. In the docking calculation, the binding site was assigned across the entire structure of TOPK, and was determined with the lowest-energy and the most favorable orientation of the ligand. Over 2924 approved drugs and nutraceuticals from ZINC Drug Database were screened in silico. Four compounds (Table 1) exhibiting significant mfScores (more negative better) and docking well into the active site of the enzyme were selected for further biological evaluation. The mfScore here represents the independent score of the strength of inhibitor-enzyme interaction. Pantoprazole was shown to have the best docking score. From the generated docking model, hydrogen bonds were predicted between the sulphone oxygen of pantoprazole with K213, phenyl ether oxygen with Y264, respectively. Also, the pyridine ring of the compound may form the π-π stacking interaction of the benzene ring of F197.

**Table 1:** Binding affinity and inhibitory activities of screening hits

Compound	ICM docking mfscore^a^ (kcal/mol)	Dissociation constant with TOPK	Inhibitory activities against HCT116 cells
		Kd^b^ (μM)	IC50 (μM)
Pantoprazole	−164	24.2± 2.3	185.8
Sulfasalazine	−142	339.0 ± 7.7	n.i ^c^
Benserazide	−86.04	477.0 ± 19.9	n.i ^c^
Practolol	−84.38	226.0 ± 29.5	n.i ^c^
HI-TOPK-032	−139.5	327.0 ± 62.7	n.p ^d^

a Docking score/interaction potential of compounds with TOPK (kcal/mol).

b The Kd value is automatic calculated by the curve fitting, and presents as means ± SD.

c n.i. is no inhibition detected in the experiments.

d n.p. is not performed in this study.

**Figure 1:**
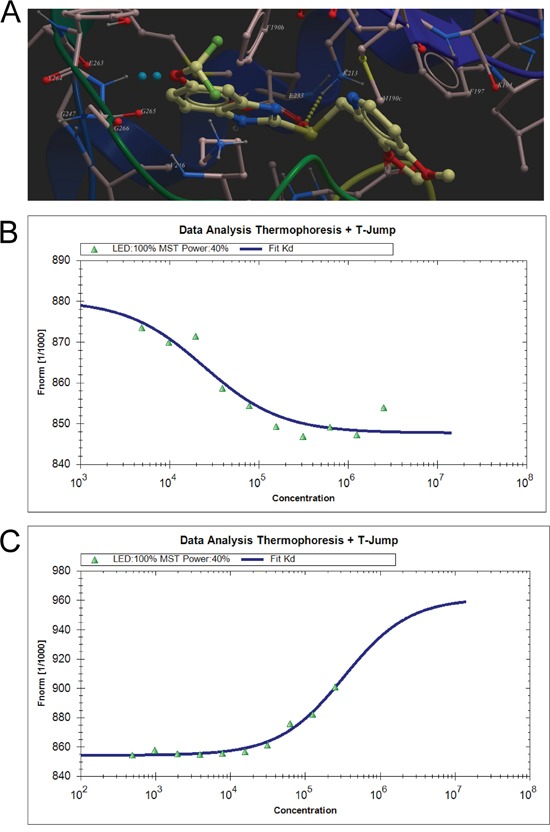
Virtual ligand screening identifies the binding of pantoprazole to TOPK **A.** Low-energy binding conformations of pantoprazole bound to TOPK generated by virtual ligand docking. The structure of TOPK was generated by homology modeling by using the structure of RIP3 kinase domain (PDB code: 4m66) as the template, and depicted in ribbon form. Pantoprazole depicted as the ball-and-stick model showing carbon (yellow), hydrogen (grey), oxygen (red), nitrogen (blue), and fluoride (green) atoms. Hydrogen bonds are represented in dotted lines. **B.** Measurement of affinity between Pantoprazole and TOPK by MST in standard treated capillaries, and the resulting binding curve was shown. From the resulting binding curve, with a Kd of 24.2± 2.32 μM. **C.** The binding curve of HI-TOPK-032 and TOPK from MST, with a Kd of 327± 63.7 μM.

To validate the finding of the virtual ligand screening, we employed microscale thermophoresis method (MST) to assay the binding affinity between the compounds and TOPK. This technology detected fluorescent changes of molecules during thermophoresis, to quantify protein-protein interactions or protein-small molecule interactions with high sensitivity and low sample cost. Among four compounds assayed, pantoprazole exhibited the lowest equilibrium dissociation constant (Kd) of 24.2± 2.32 μM (Table 1, Figure 1A, 1B, Supplementary Figure 1A, 1B, 1C), which meant the strongest binding. The binding of pantoprazole was also stronger than that of HI-TOPK-032, a previously reported TOPK inhibitor (Table 1, Figure 1C).

### Pantoprazole inhibits EGF-induced anchorage-independent growth of JB6 Cl41 cells

**Figure 2:**
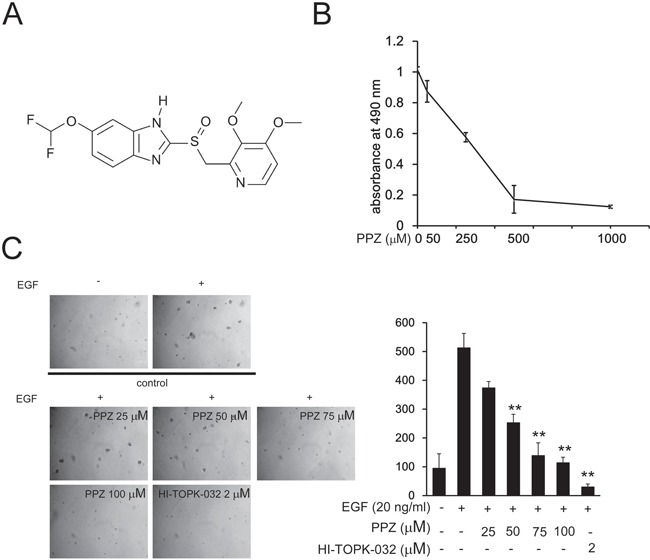
Pantoprazole inhibits EGF-induced anchorage-independent growth of JB6 Cl41 cells **A.** The chemical structure of pantoprazole. **B.** Cytotoxic effects of pantoprazole on mouse epidermal JB6 Cl41 cells. An MTS assay was used after treatment of cells with pantoprazole for 24h. **C.** Pantoprazole inhibits EGF-induced anchorage-independent growth of JB6 Cl41 cells. JB6 Cl41 cells (8 × 10^3^) were exposed to EGF (10 ng/ml) and treated with pantoprazole (0-50μM) in 1 mL of 0.3% Basal Medium Eagle (BME) agar containing 10% FBS, 2 mM L-glutamine, and 25 μM gentamicin. The cultures were maintained at 37°C, in a 5% CO_2_ incubator for 14 days, and the cell's colonies were scored using a microscope Motic AE 20 (China) and the Motic Image Plus computer program. The numbers of colonies represent the whole culture well colonies. Data are shown as mean ± standard deviation from triplicate experiments. The P values indicate a significant inhibition by pantoprazole in colony formation (*P < 0.05; **P < 0.01).

The chemical structure of pantoprazole is shown in Figure 2A. To determine the cytotoxicity of pantoprazole, different concentrations of the drug were used to treat JB6 Cl41 mouse epidermal cells (JB6 Cl41cells) for 24 h. Cytotoxicity was measured by MTS assay and the results indicated that pantoprazole had no cytotoxicity toward JB6 Cl41 cells up to 100 μM at 24 h (Figure 2B). Next, we investigated whether pantoprazole had an effect on anchorage-independent growth of JB6 Cl41 cells. Anchorage-independent growth ability is a hallmark of *in vitro* transformed cells and cancer cells. Our results revealed that JB6 Cl41 cells treated with pantoprazole formed fewer colonies compared with the control cells treated with only epidermal growth factor (EGF) (Figure 2C). These results showed that pantoprazole could attenuate EGF-induced anchorage-independent cell growth and was not cytotoxic in clinically relevant doses.

### Pantoprazole suppresses TOPK activity *in vitro* and in JB6 Cl41 cells

**Figure 3:**
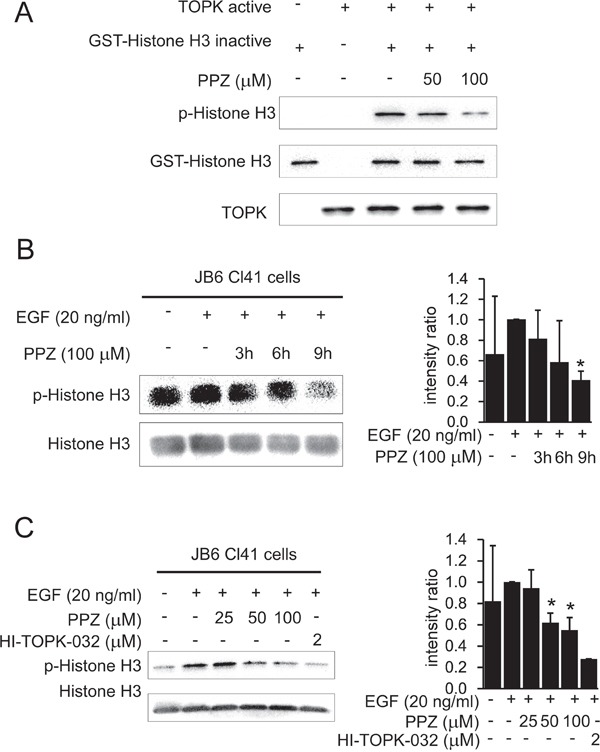
Pantoprazole suppresses TOPK activity *in vitro* and in JB6 Cl41 cells **A.** Pantoprazole inhibits TOPK activity *in vitro*. The inhibitory effect of pantoprazole on TOPK was determined by an *in vitro* kinase assay as described in section ‘Materials and methods’. The expression level of phosphorylated histone H3 (Ser10) was confirmed by western blot analysis using an antibody against phosphorylated histone H3. GST-histone H3 was used as a loading control. Data are representatives of results from triplicate experiments. **B.** Pantoprazole inhibits TOPK activity JB6 Cl41 cells. Cells were treated in the presence or absence of 100μM pantoprazole for various times, then treated with 20 ng/ml EGF for 30 minutes, histones were extracted from cells, electrophoresed on 15% SDS-PAGE gels, total histone H3 and phosphorylated histone H3 proteins were detected by western blot using specific antibodies. Data are representatives of results from triplicate experiments and the intensity ratio of p-Histone H3 to total Histone H3 was calculated by an image J software. **C.** Different concentrations of pantoprazole were incubated with JB6 cells for 9 h and 2 μM of HI-TOPK-032 was a positive control.

Based on our previous data showing that pantoprazole directly binds with TOPK, we adopted an *in vitro* kinase assay with histone H3 as substrate to further confirm that pantoprazole inhibits TOPK activities. The results showed that phosphorylation of histone H3 (Ser10) was substantially attenuated in a dose-dependent manner after treatment with pantoprazole (Figure 3A). In addition, we examined whether pantoprazole could suppress TOPK activities in JB6 Cl41 cells. Data indicated that phosphorylation of histone H3 (Ser10) was lessened by treatment with pantoprazole in a time-dependent (Figure 3B) and dose-dependent manner (Figure 3C).

### Pantoprazole inhibits anchorage-independent growth of colorectal cancer cells

**Figure 4:**
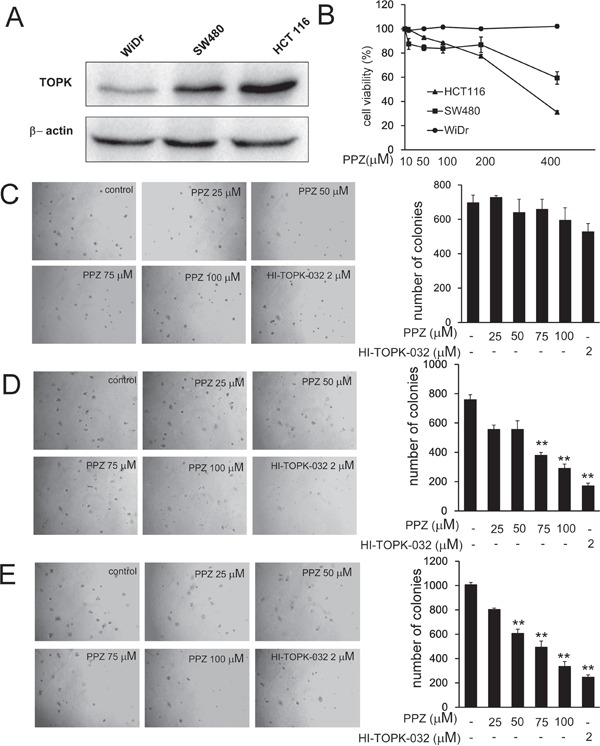
Pantoprazole inhibits anchorage-independent growth of colorectal cancer cells **A.** Expression of TOPK in colon cancer cell lines WiDr, SW480, and HCT 116. **B.** Different concentrations of pantoprazole were used to treat colon cancer cell lines WiDr, SW480, and HCT 116 for 48h respectively. Cytotoxicity was measured by MTS assay. **C-E.** The effect of pantoprazole on anchorage-independent growth of colon carcinoma cell lines with different level of TOPK expression, including WiDr cells (C), SW480cells (D), and HCT 116 cells (E). WiDr cells, SW480cells, and HCT 116 cells (8 × 10^3^ cells/mL) were treated with pantoprazole (0 - 100 μM) in 1 mL of 0.3% Basal Medium Eagle (BME) agar containing 10% FBS, 2 mM L-glutamine, and 25 μg/mL gentamicin. The cultures were maintained at 37°C, in a 5% CO_2_ incubator for 14 days, and the cell's colonies were scored using a microscope Motic AE 20 (China) and the Motic Image Plus computer program. The numbers of colonies represent the whole culture well colonies. Data are shown as means ± standard deviation of values from three independent experiments and the asterisk indicates a significant (*p < 0.05, **p < 0.01, ***p < 0.001) decrease in colony formation in cells treated with pantoprazole compared with the PBS-treated group.

Previous studies suggested that TOPK is highly activated in human colon cancer. We attempted to determine whether pantoprazole could affect anchorage-independent growth of colon cancer cells. We used three colon cancer cell lines HCT 116, SW480 and WiDr with high, middle and low expression level of TOPK, respectively (Figure 4A). To determine the cytotoxicity of pantoprazole, different concentrations of the drug were used to treat colon cancer cell lines HCT 116, SW480 and WiDr for 48h, respectively (Figure 4B). Cytotoxicity was measured by MTS assay and the results indicated that pantoprazole had different cytotoxicities toward different colon cancer cells. HCT 116 cells with high level TOPK were more sensitive to pantoprazole (Figure 4E). The cells were maintained with different concentrations of pantoprazole and colony numbers were counted after culturing for 7-14 days. The results showed that pantoprazole at 25, 50, 75, 100 μM and HI-TOPK-032 at 2 μM inhibited colony formation of WiDr on 0, 8, 5, 15 and 24%; SW480 cells on 27, 27, 50, 62 and 77% and HCT 116 on 20, 40, 51, 67 and 75% respectively, compared with the non-treated cells (Figure 4C-4E). Overall, our results suggested that inhibitory effect of pantoprazole on colony formation was significant in HCT 116 cells with a high expression level of TOPK.

### Knocking down TOPK attenuates the inhibitory effect of colon cancer cell growth by pantoprazole

**Figure 5:**
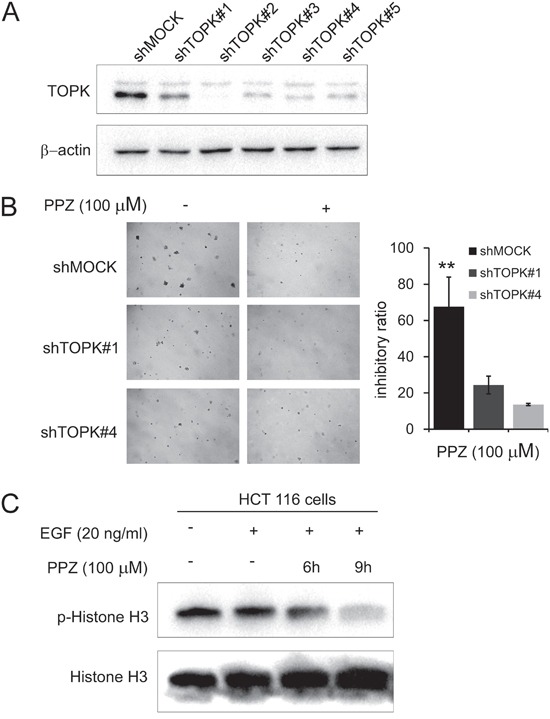
Knocking down TOPK attenuates the inhibitory effect of colon cancer cell growth by pantoprazole **A.** Expression level of TOPK in HCT116 cells is decreased by knockdown of TOPK. HCT116 cells were transiently transfected with shMOCK or shTOPK (#1-#5) and cell lysates were analyzed by western blot. **B.** TOPK has less effect on anchorage-independent cell growth of shTOPK transfected cells than that of shMOCK cells. HCT116 cells were grown in soft agar with pantoprazole (100 μM) for 10 days and the colonies were counted. Data are represented as mean ± standard deviation from triplicate experiments. The asterisks (**) indicate a significant decrease compared with shMOCK cells (*P* < 0.01). **C.** Pantoprazole inhibits TOPK activity in HCT 116 cells. After HCT116 cells (2 × 10^6^) were cultured in a 10-cm dish for 24h, the cells were starved in serum-free medium for another 24h, in the meantime the cells were treated with pantoprazole (100 μM) for (0-12 h), then treated with EGF (80 ng/ml) for 15 min. The cells were then harvested and the protein levels were determined by western blot analysis using specific antibodies. Data are representatives of results from triplicate experiments.

In addition, to investigate whether the effects of pantoprazole are mediated directly through TOPK, we compared the effects of HCT 116 cells transfected with a shMOCK or shTOPK plasmid (#1-#5) (Figure 5A). Pantoprazole suppressed anchorage-independent growth in shMOCK cells but had less effects in shTOPK cells (Figure 5B). Western blot results showed that TOPK-mediated phosphorylation of histone H3 (Ser10) was substantially decreased time dependently with pantoprazole treatment (Figure 5C). These results suggested that TOPK is a direct target for pantoprazole to suppress colon cancer cell growth.

### Pantoprazole suppresses tumor growth by inhibiting TOPK activity *in vivo*

**Figure 6:**
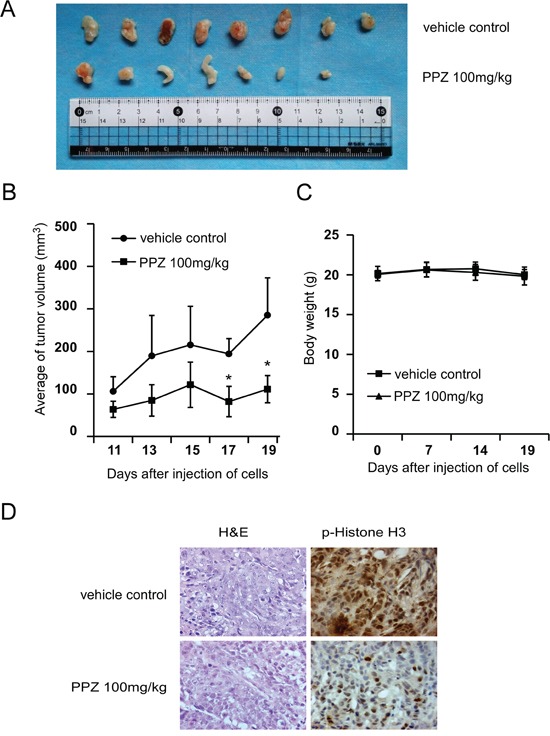
Pantoprazole suppresses tumor growth by inhibiting TOPK activity *in vivo* **A.** Pantoprazole significantly suppresses cancer growth in a HCT116 xenograft mouse model. **B.** The average tumor volume of vehicle-treated control mice (n=8) and pantoprazole treated mice (n=8) plotted over 20 days after tumor cell injection. The asterisk (*) indicates a significant increased tumor size (*P* < 0.05) in the vehicle-treated group compared with that in the pantoprzole-treated group as determined by one-way analysis of variance. **C.** Pantoprazole has no effect on mouse body weight. Body weights from the treated or untreated groups of mice were measured once a week. **D.** Pantoprazole inhibits expression of phosphorylated histone H3 *in vivo*. Immunohistochemistry analysis was used to determine the level of phosphorylated histone H3 in tumor tissues.

Based on our previous data, the next step was to determine whether pantoprazole could suppress tumor growth *in vivo*. After injecting HCT 116 cells into nude mice, and then the mice were divided into two matched groups. Treatment was started after two days of cells injection. The first measurable tumors were observed on day 11. The results indicated that tumors treated with 100 mg/kg pantoprazole grew significantly more slowly and the size of tumors was smaller compared with the vehicle group (Figure 6A). On day 19, the average tumor volume per mouse treated with 100 mg/kg pantoprazole was 111mm^3^ while that was 285mm^3^ in the vehicle group (Figure 6B). The average body weights of either group were similar throughout the study (Figure 6C), which indicated that the dose of pantoprazole used for the experiment had no toxicity to the mice. To further determine whether the antitumor effect of pantoprazole was associated with its inhibition of TOPK activities, tumor extracts from either group were prepared and analyzed for phosphorylation of histone H3. The results of immunohistochemistry analysis showed that expression of phosphorylated histone H3 was substantially decreased in the pantoprazole-treated group compared with the vehicle group (Figure 6D). Overall, our results indicated that pantoprazole suppressed tumor growth by inhibiting TOPK activities *in vivo*.

## DISCUSSION

Pantoprazole, an FDA-approved proton-pump inhibitor, herein is reported to exert antitumor activities by targeting TOPK. TOPK inhibitors which could benefit 30-40% of CRC patients might represent a new avenue of investigation for targeted therapy [16]. Therefore, it is important to find a TOPK inhibitor that could be put into clinical use as soon as possible.

In the process of new drug development, it is difficult to move from preclinical screening to clinical trials for its high cost and low success rate. Drug repurposing is the process of exploiting new indications for existing drugs [17]. The supposition of drug repositioning is that these types of drugs are probably to enter clinical trials faster and less expensively, because of verified bioavailability and compatibility. Thus, interests in this strategy have been growing fast in recent years. For example, Orlistat, an FDA-approved anti-obesity drug, was found to inhibit endothelial cell proliferation and angiogenesis by suppressing several new targets. As a result, this compound, as well as other orlistat like analogues, has been proposed as potential anticancer drugs [18].

Structure based virtual ligand screening has emerged as an effective way to find lead compounds for given targets in a high-throughput manner with low cost, while avoiding purchasing thousands of compounds before obtaining hits [19–22]. The combination of structure-based virtual ligand screening and drug repositioning thus represents an efficient method to find new drugs. In order to find out new inhibitors of TOPK that might be applied clinically immediately, we performed structure based virtual ligand screening against ZINC Drug Database by a homology model of human TOPK. Over 2924 approved drugs and nutraceuticals from ZINC Drug Database were screened and four drugs were found to be potential inhibitors. We further confirmed that pantoprazole, a FDA-approved proton-pump inhibitor (PPI), was an effective TOPK inhibitor. Pantoprazole bound specifically with TOPK and inhibited its activities. The binding between pantoprazole and TOPK was even stronger than that with the known TOPK inhibitor HI-TOPK-032. Compared with HI-TOPK-032 and OTS964, pantoprazole has much lower cytotoxicities (Supplementary Figure 2). All these tend to imply that pantoprazole is at least a good lead compound for designing novel TOPK inhibitors.

Pantoprazole is a standard treatment for gastric acid-related diseases and Helicobacter pylori infection together with antibiotics [23]. Besides targeting the gastric acid pump, PPIs inhibit activities of V-ATPase [24]. Recent studies reported that the pretreatment of PPIs increased sensitivities of drug-resistant cancer cells to cytotoxic drugs, such as melanomas [25], lymphomas [26] and gastric adenocarcinomas [27, 28]. There are also some data providing the proof of the concept that PPIs may be used as antineoplastic agents, but the underlying mechanisms is far from conclusive. Pantoprazole as a proton-pump inhibitor, is a prodrug, which requires protonation for functional activation under acidic conditions, accumulated selectively in acidic gastric luminal space, and ultimately inhibits acid secretion by covalently binding to cysteine residues in α-subunit of H^+^/K^+^-ATPase [29–30]. However, in this study, cells were treated in medium with pH 7.2-7.4, not in acid condition. It is strongly implied that pantoprazole inhibits tumor growth by its prodrug form.

Previous studies showed that TOPK was a novel serine-threonine kinase that was a member of MAPKK family. TOPK was involved in many cellular functions, including tumor development, cell growth, apoptosis, and inflammation [31–33]. During mitosis, kinase activities of TOPK is elevated, and TOPK promotes phosphorylation of histone H3, PRC1 (protein regulator of cytokinesis 1), GPSM2 [G protein (heterotrimeric guanine nucleotide-binding protein) signaling modulator 2], and p97 proteins that are essential for the completion of cancer cell cytokinesis [34–36]. Therefore, it is likely that TOPK phosphorylates various protein substrates that are essential for cancer cell survival and proliferation, particularly in the mitotic phase of cell cycle.

Consistent with reported studies, our results indicated that the phosphorylation of histone H3 (Ser10) *in vitro* and *in vivo* were both strongly reduced by pantoprazole (Figure 3A-3C, Figure 5C). Notably, the phosphorylation of histone H3 (Ser10) was inhibited *in vivo* in pantoprazole–treated tumor tissues (Figure 6D).

In conclusion, we provided evidences showing that pantoprazole effectively suppressed anchorage-independent cell growth of colon cancer cells with high expressed TOPK levels, and suppressed *in vivo* tumor growth of HCT 116 cells by inhibiting TOPK activities. Overall, our findings offer an alternative therapy for colorectal cancer by targeting TOPK with pantoprazole.

## MATERIALS AND METHODS

### Reagents and antibodies

Pantoprazole sodium for injection was purchased from Hangzhou Zhongmei Huadong Pharmaceutical Co., Ltd (China). MTS (3-(4,5-dimethylthiazol-2-yl)-5-(3-carboxymethoxyphenyl)-2H-tetrazdium) assay kit was purchased from “Promega” (USA). Antibodies against phospho-Histone H3, TOPK/PBK, Histone H3, were obtained from “Cell Signaling Technology” (USA), β-actin and horseradish peroxidase (HRP) conjugated secondary antibody from rabbit and mouse were purchased from “Santa Cruz” (USA) and “Protein Tech Group” (USA), respectively. The chemiluminescence's detection kit ECL Plus was from “GE Healthcare” (USA). The Basal Medium Eagle (BME), Dulbecco's Modified Eagle medium (DMEM), Minimum Essential medium (MEM), McCoy's 5A Modified medium (McCoy's 5A), Roswell Park Memorial Institute medium (RPMI 1640), phosphate buffered saline (PBS), L-glutamine, gentamicin solution, trypsin, fetal bovine serum (FBS), sodium hydrocarbonate (NaHCO_3_), and agar were purchased from “Sigma” and “Gibco” (USA). All other common chemicals, solvents and reagents were of highest grade available from various commercial sources.

### Cell lines and culture conditions

Mouse epidermal JB6 Cl41 cells (ATCC # CRL-2010) and human colon cancer cells HCT 116(ATCC # CCL-247™), SW480 (ATCC # CCL-228™), WiDr (ATCC # CCL-218™) were obtained from the American Type Culture Collection (ATCC, USA). Mouse epidermal JB6 Cl41, human colon cancer HCT 116, SW480, and WiDr cells were cultured in MEM/5% FBS, McCoy's 5 A/10% FBS, RPMI-1640/10% FBS and RPMI-1640/10% FBS media, respectively. The cell cultures were maintained at 37°C in humidified atmosphere containing 5% CO_2_. The cells were grown for 3-4 days, and after reaching 90% of confluence they were harvested by exposure to 0.25% Trypsin-EDTA or 0.25% Trypsin without EDTA (JB6 Cl41) solution and then passed into new T-75 tissue culture flasks. The cell cultures were performed following the instructions of ATCC.

### Microscale thermophoresis (MST)

Recombinant TOPK was labeled with the Monolith NT™ Protein Labeling Kit RED (Cat#L001) according to the supplied labeling protocol. Labeled TOPK was used in a concentration of 50 nM. The samples were diluted in a 20 mM HEPES (pH 7.4) and 0.05 (v/v) % Tween-20. The pantoprazole stock was dissolved in ddH_2_O in a concentration of 5 mM. We used 5mM pantoprazole as the highest concentration for the serial dilution. After 10 minutes incubation at room temperature the samples were loaded into MonolithTM standard-treated capillaries and the thermophoresis was measured at 25°C after 30 minutes incubation on a Monolith NT.115 instrument (NanoTemper Technologies, München, Germany). Laser power was set to 20% or 40% using 30 seconds on-time. The LED power was set to 100%. The dissociation constant Kd values were fitted by using the NTAnalysis software (NanoTemper Technologies, München, Germany) [37].

### Homology modeling and molecular docking

With the crystal structure of the IRAK-4 kinase (PDB code: 2NRU) as the template, a homology model of human TOPK (accession number: NP_060962) was constructed by using MODELLER (an automated homology modeling program) [38–39]. The subset zdd (ZINC Drug Database), which included all commercially available approved drugs and nutraceuticals worldwide, was downloaded from ZINC as mol2 files, which were used as input for docking [40]. The docking was performed by using ICM 3.8.1 modeling software on an Intel i7 4960 processor (MolSoft LLC, San Diego, CA) [41]. Ligand binding pocket residues were selected by using graphical tools in the ICM software, to create the boundaries of the docking search. In the docking calculation, potential energy maps of the receptor were calculated using default parameters. The compounds were imported into ICM and an index file was created. The conformational sampling was based on the Monte Carlo procedure, and finally the lowest-energy and the most favorable orientation of the ligand was selected.

### MTS assay

To estimate cell viability, mouse epidermal JB6 Cl41, human colon cancer HCT 116, SW480, and WiDr cells (1000/well) were seeded in 96-well plates for 24 h at 37°C in a 5% CO_2_ incubator. The attached cells were fed with fresh medium containing various concentrations of pantoprazole (0-100 μM) for additional 24 h and 48 h. After culturing for various times, the cytotoxicity of pantoprazole was measured using an MTS assay kit according to the manufacturer's instructions. All the experiments were performed in triplicate, and the mean absorbance values were calculated. The results are expressed as the percentage of inhibition that produced a reduction in absorbance by pantoprazole treatment compared with the non-treated cells (control).

### Anchorage-independent cells transformation assay (Soft agar)

Mouse epidermal JB6 Cl41 cells (2.4 × 10^4^) were exposed to EGF (20 ng/ml) and treated with pantoprazole (0-100 μM) in 1 mL of 0.3% Basal Medium Eagle (BME) agar containing 10% FBS, 2 mM L-glutamine, and 25 μg/mL gentamicin. The cultures were maintained at 37°C, in a 5% CO_2_ incubator for 7-14 days, and the cell's colonies were scored using a microscope Motic AE 20 (China) and the Motic Image Plus computer program. To estimate the effect of pantoprazole on colony formation, colon cancer cells HCT 116, SW480, and WiDr (2.4 × 10^4^) were treated with pantoprazole (0-100 μM) in 1 mL of 0.3% Basal Medium Eagle (BME) agar containing 10% FBS, 2 mM L-glutamine, and 25 μg/ml gentamicin. The cultures were maintained in a 37°C, 5% CO_2_ incubator for 7-14 days, and the cell colonies were scored as described above.

### *In vitro* kinase assay

Inactive histone H3 proteins were used as the substrate for an *in vitro* kinase assay with active TOPK. Firstly, active TOPK was incubated with pantoprazole (50 and 100 μM) in 1 × kinase buffer (25 mM Tris-HCl pH 7.5, 5 mM b-glycerophosphate, 2 mM dithiothreitol (DTT), 0.1 mM Na_3_VO_4_, 10 mM MgCl_2_, and 5 mM MnCl_2_) at 32°C for 15 minutes. Then inactive histone H3 and 100 μM ATP were added to reaction and incubated at 32°C for 1.5 h. Reactions were stopped by adding 5 × SDS sample buffer and then were analyzed by Western blot.

### Western blot analysis

The harvested cells were lysed with lysis buffer (50 mM Tris-HCl (pH 7.4), 150 mM NaCl, 1 mM EDTA, 1 mM EGTA, 10 mg/mL aprotinin, 10 mg/mL leupeptin, 5 mM phenylmethanesulfonyluoride (PMSF), 1 mM dithiolthreitol (DTT) containing 1% Triton X-100). Insoluble debris was removed by centrifugation at 12000 rpm for 15 minutes, and protein's content was determined using Bradford reagent (Bio-Rad, USA). Lysate protein (20-40 μg) was subjected to 10% SDS-PAGE and electrophoretically transferred to polyvinylidene difluoride membranes (PVDF) (Millipore, USA). The membranes were blocked with 5% non-fat milk or 5% BSA for 1 h and then incubated with the respective specific primary antibody at 4°Covernight. Protein bands were visualized using an enhanced chemiluminescence reagent (ECL Plus) (GE Healthcare, USA) after hybridization with a HRP conjugated secondary antibody. Band density was quantified using the Image J software program (NIH).

### Isolation of histone H3

Histones were extracted from cells by disrupting the cells with NETN buffer [150 mM NaCl, 1 mM EDTA, 20 mM Tris-HCl (pH 8.0), 0.5% nonionic detergent IGEPAL CA 630(NP-40), Sigma]. The insoluble fraction was pelleted for 5 minutes in a microcentrifuge (8,400 rpm). Nuclei were extracted with 0.1 M HCl to isolate the total histones. The samples were precipitated with 1 M Tris-HCl (pH 8.0) and then resuspended in ddH_2_O.

### *In vivo* xenograft mouse model

Non-obese Diabetic/Severe Combined Immunodeficiency (NOD-SCID) mice were purchased from Beijing HFK Bioscience CO., LTD (Beijing, China). The animals were maintained under ‘specific pathogen-free’ conditions according to the guidelines established by Research Animal Resources, Laboratory Animal Center, The Fourth Military Medical University (China). The mice were randomly divided into two groups: (i) vehicle group (n=8); (ii) 100 mg/kg pantoprazole-treated group (n=8). The experiment was repeated with HCT 116 colon cancer cells. HCT 116 cells were inoculated subcutaneously (5×10^6^ cells) into the left flank of each mouse in the two groups. Treatment was started after two days of cells injection. For the pantoprazole group, 2.5 mg pantoprazole, formulated in 200 μl physiological saline, was administered to each mouse every two days by intraperitoneal (i.p.) injection. For the vehicle group, 200 μl physiological saline was administered to each mouse every two days by i.p. injection. The duration of the animal study was 19 days for HCT 116 cells. The tumor volume was calculated from measurements of 3 diameters of the individual tumor based on the following formula: tumor volume (mm^3^) = (length × width × height × 0.52). The mice were monitored until tumors reached 1 cm^3^ total volume, at which time the mice were euthanized and the tumors were extracted. The tumors were dissected and sent for immunohistochemical analysis at the Department of Pathology in Xijing Hospital (The Fourth Military Medical University, China). All animal experiments were performed following the protocols approved by the Laboratory Animal Center of the Fourth Military Medical University.

## SUPPLEMENTARY FIGURES


