# Supplementary material for: Pantoprazole, an FDA-approved proton-pump inhibitor, suppresses colorectal cancer growth by targeting T-cell-originated protein kinase
Source: Oncotarget. 2016 Mar 8;7(16):22460–73. doi: 10.18632/oncotarget.7984 (PMC5008373; doi:10.18632/oncotarget.7984)
Supplement: Supplementary file 1 [file oncotarget-07-22460-s001.pdf]

SUPPLEMENTARY FIGURES

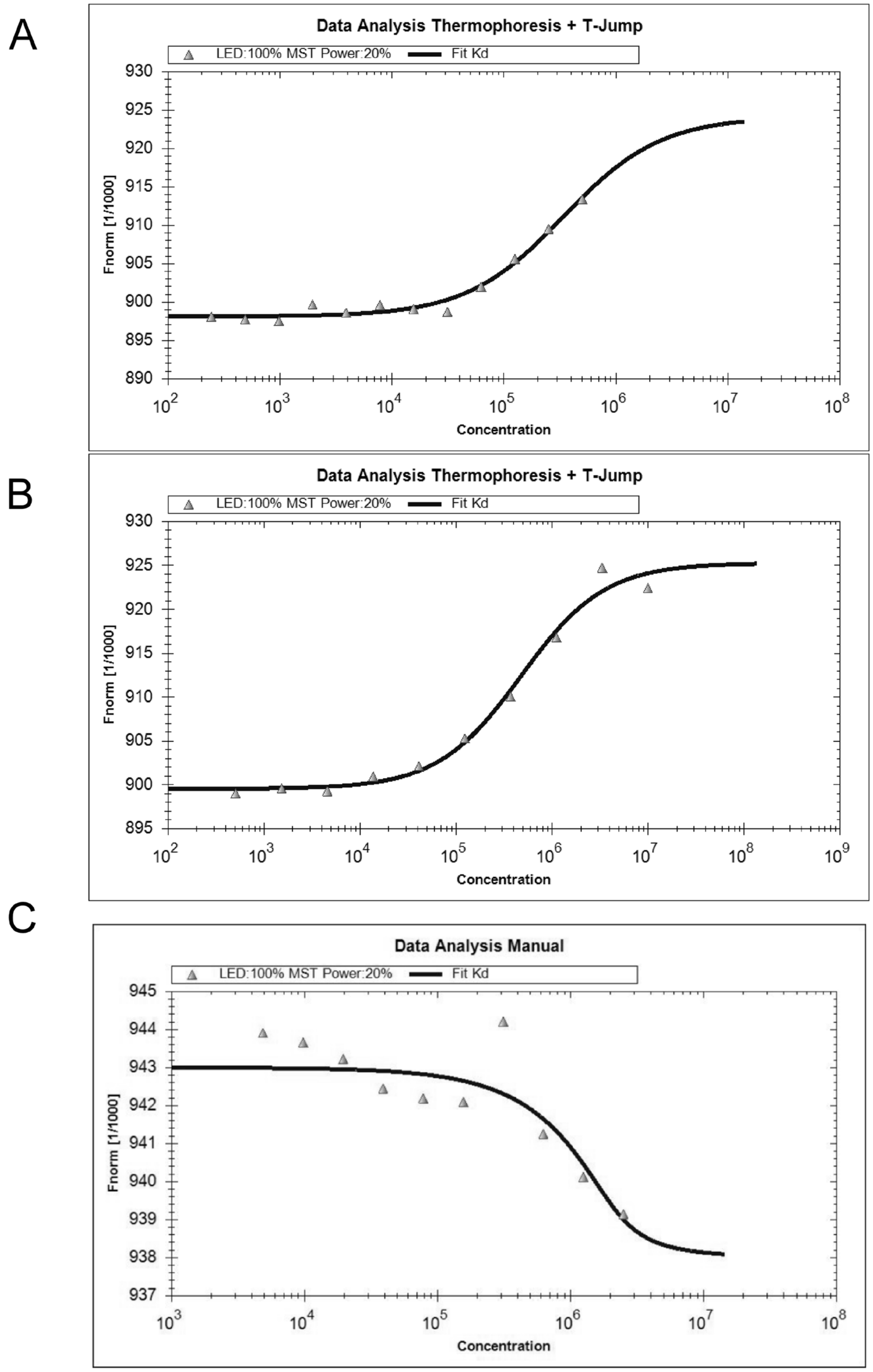

Supplementary Figure S1: Measurement of affinity between screen hits and TOPK by MST. A. Measurement of affinity between sulfasalazine and TOPK by MST in standard treated capillaries, the resulting binding curve was shown. From the resulting binding curve, with a Kd of 339.0 ± 7.7μM. **B.** The binding curve of benserazide and TOPK from MST, with a Kd of 477.0 ± 19.9μM. **C.** The binding curve of practolol and TOPK from MST, with a Kd of 226.0 ± 29.5μM.

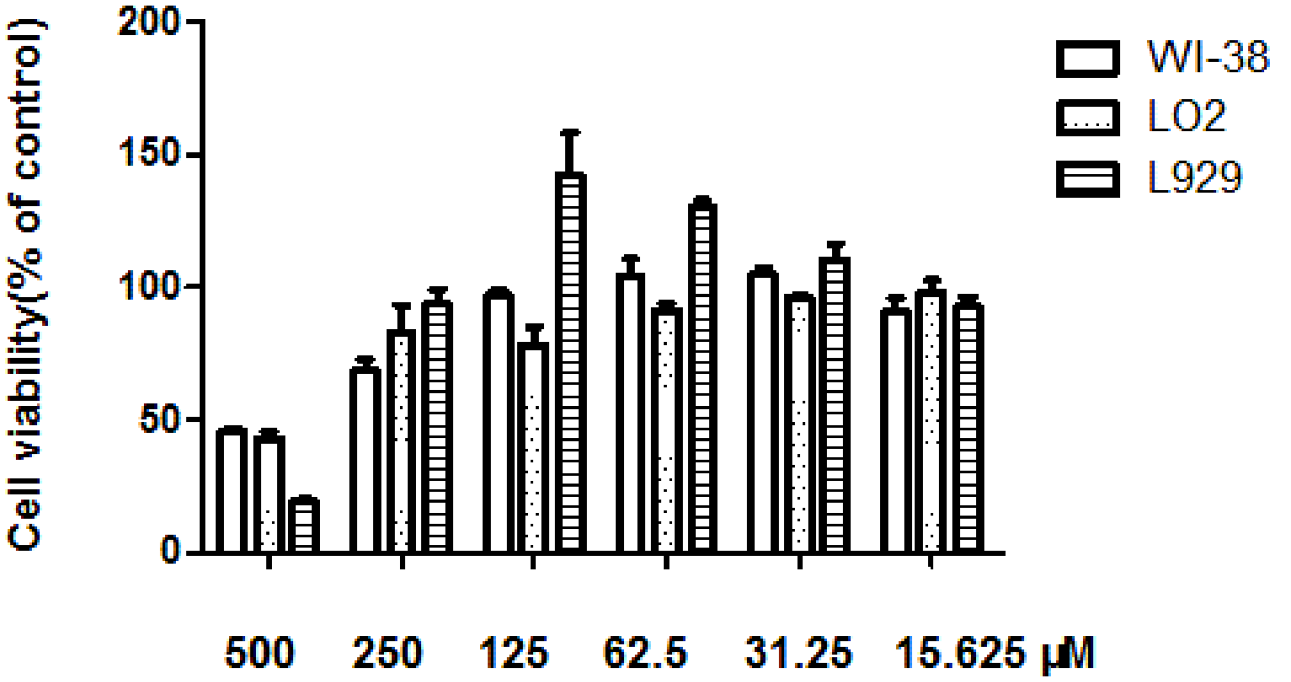

Supplementary Figure S2: Cytotoxicity of pantoprazole on cell viability as measured by the MTT method. The WI-38 cells, LO2 cells and L929 cells were treated with the indicated concentrations of pantoprazole for 72 h. The data are indicated as the percentage of living cells compared with the vehicle control. Results are representative of three independent experiments. The IC50 of WI-38 cells, LO2 cells and L929 cells were 432.8 ± 16.8µM, 479.0 ± 25.3µM and 397.5 ± 35.6µM, respectively.
